# Retinal and choroidal thickness measurements using spectral domain optical coherence tomography in anterior and intermediate uveitis

**DOI:** 10.1186/1471-2415-14-103

**Published:** 2014-08-30

**Authors:** Zsuzsanna Géhl, Kinga Kulcsár, Huba JM Kiss, János Németh, Otto A Maneschg, Miklós D Resch

**Affiliations:** 1 Department of Ophthalmology, Semmelweis University Budapest, Mária u. 39, Budapest H-1085, Hungary

**Keywords:** Anterior uveitis, Enhanced depth imaging, Intermediate uveitis, Macular edema, Optical coherence tomography

## Abstract

**Background:**

Macular edema is a common cause of visual loss at uveitic patients. The aim of our study was to investigate retinal and choroidal thickness at the macula in anterior (AU) and intermediate (IMU) uveitis and in healthy individuals using spectral domain optical coherence tomography (SD-OCT).

**Methods:**

Case-control study of 21 patients with AU and 23 patients with IMU and 34 age-matched healthy controls was performed with Spectralis SD-OCT (Heidelberg Engineering, Germany). High resolution SD-OCT scans and macular mapping were applied for automated measurement of retinal thickness. Standardized, masked manual measurement of the choroidal thickness was performed in the center of the ETDRS fields on enhanced depth imaging (EDI) scans. Evaluation of central retinal subfield thickness, 3 mm and 6 mm perifoveal rings was performed in the corresponding ETDRS zones in patient groups.

**Results:**

The mean central retinal subfield thickness was significantly higher in IMU (368.65 ± 115.88 μm, p = 0.0003), but not significantly different in AU (290.42 ± 26.37 μm p = 0.6617) compared to that of in controls (278.55 ± 18.31 μm). In both uveitis groups retina was significantly thicker in the 3 and 6 mm perifoveal rings than that of in controls (359 ± 15.24 μm in AU and 390.55 ± 70.90 μm in IMU vs 345,41 ± 15.28 μm in the control group, p = 0.0388 and p < 0.0001) in the 3 mm and (313.83 ± 16.63 μm in AU and 343.33 ± 57.29 μm in IMU vs 299 ± 13.82 μm in the control group, p = 0.0171 and p < 0.0001) in the 6 mm ring. Central choroidal thickness was 311.94 ± 60.48 μm in the control eyes, showed no significant difference in AU (312.61 ± 90.35 μm) and IMU (303.17 ± 93.66 μm) eyes, and was also similar at the perifoveal rings.

**Conclusion:**

Significant topographical changes could be detected in the macula of AU and IMU patients. Retinal thickness in the perifoveal rings was increased both in AU and IMU, but in the center only in IMU. Choroidal thickness seems to be unaffected by uveitis, even in the presence of macular edema, at least in the early stage of the inflammatory disease process.

## Background

Anterior uveitis (AU) is the most common type of uveitis (49%) and the proportion of intermediate uveitis (IMU) is also estimated to be above the 10% threshold in referral centers [1,2]. According to the Standardization of Uveitis Nomenclature Working Group (SUN), in AU the primary site of inflammation is the anterior chamber and in IMU it is the vitreous. In the background of both types of uveitis numerous etiological causes are recognized [3].

Macular edema is the most common cause of permanent visual loss in IMU. Nevertheless, macular edema may occur in any type of ocular inflammation, among others in AU as well [2,4,5]. The presence of cystoid macular edema in AU is not uncommon, but especially in the early inflammatory process it evolves into only diffuse macular thickening [2,4]. Early recognition of macular edema is important in the visual outcome. If persisting for several months, macular edema gives rise to irreversible photoreceptor damage leading to permanent loss of central vision. With delayed treatment of macular edema only limited visual recovery can be achieved [6].

Subclinical macular edema may be easily misdiagnosed by performing biomicroscopy, but with the help of optical coherence tomography (OCT) macular edema can be detected already in an early phase [4,5]. Recently the introduction of the spectral-domain OCT (SD-OCT) improved not only retinal image resolution, but some instrumental setups now allow a better visualization of the choroid as well. The Spectralis (Heidelberg Engineering, Heidelberg, Germany) incorporating software, with its enhanced depth imaging (EDI) technology allows for the good quality imaging of the choroid, permitting the qualitative and quantitative analysis of this layer [7]. EDI-ready devices gained great significance in the detection of inflammatory processes involving the choroid.

The aim of the present study was to determine the retinal and choroidal thickness in the macula in AU and IMU patients. In AU and IMU macular edema can be a structural complication affecting the posterior segment [3]. Further aim of the study was to correlate retinal and choroidal changes in uveitic macular edema.

## Methods

A retrospective analysis was performed on 44 eyes of 44 consecutive uveitic patients (21 AU and 23 IMU eyes) and 34 eyes of 34 healthy subjects, who underwent OCT examination using Heidelberg Spectralis SD-OCT between January 2012 and January 2013 at the Department of Ophthalmology at Semmelweis University. The study was approved by the local Ethics Committee (ETT TUKEB) and was conducted in accordance with the Declaration of Helsinki. One eye per patient was selected as the study eye. If the inflammation was bilateral, the eye with better visualization of the choroid–scleral junction on OCT images was chosen.

Normal subjects had 20/20 best corrected visual acuity with a refractive error level below +6.0 or -6.0 diopters, and they did not have any ocular pathology or any ophthalmic surgery in the history.

Inclusion criteria of uveitic patients were: active biomicroscopic signs of AU or IMU. OCT examination was performed on the first visit of the patient with newly diagnosed uveitis at our uveitis center, or on the first visit of the patients with recurrence after a quiescent period. The anatomic localization of the uveitis was established according to SUN criteria [3]. After detailed medical history and slit lamp examination standard diagnostic work up (laboratory examination including complete blood counts, comprehensive metabolic panel, erythrocyte sedimentation rate, C-reactive protein, chest x-ray), and in selected cases with the suspect of specific etiology: QuantiFERON-TB Gold, angiotensin-convertase enzyme, immune panel, sacroiliacal x-ray, brain MRI, chest CT, and specific serological assays were performed regarding herpes viruses, lues, toxoplasmosis, bartonella and borrelia.

In this series we had three patents in AU group with rheumatological signs and HLA-B27 positivity. Either in AU or in IMU group patients with other proved specific and systemic etiology, and infectious origin were excluded.

Further exclusion criterion was vitreoretinal traction or epiretinal membrane causing significant retinal distortion. Uveitis was in the early period of the inflammatory process in all patients. The OCT imaging was made at the first visit of the first or the recurrent inflammatory period, within 1-3 weeks, as far as possible we could tell from the patients history. In these cases the vitreous haze was maximum 2+ severity.

### OCT acquisition and analysis protocol

**Figure 1:**
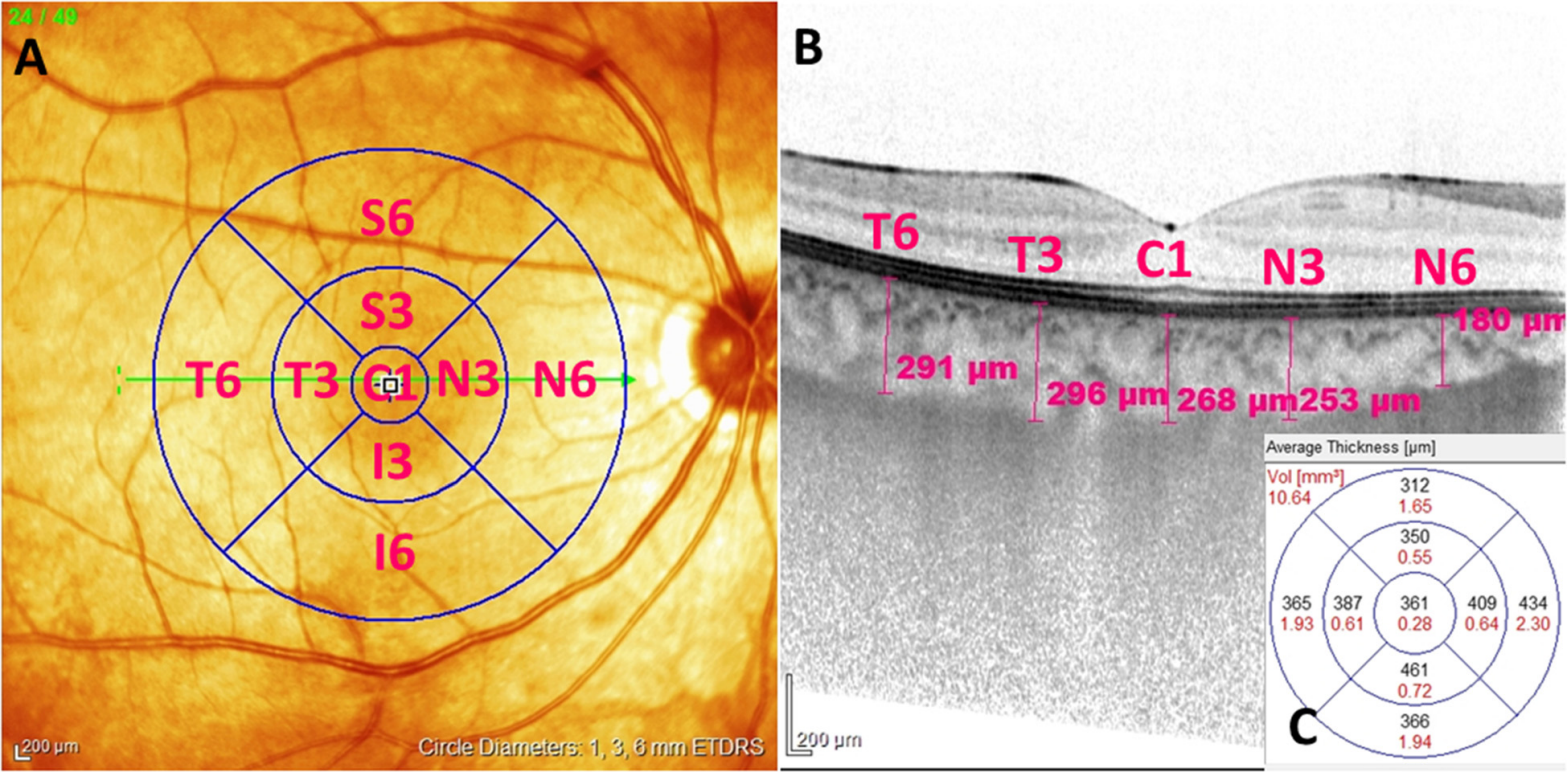
**Measurement of choroidal and retinal thickness in ETDRS fields. 1A**. Demonstrates the 1, 3 and 6 mm diameter ETDRS circles superimposed on the pseudocolor infrared image. The horisontal line shows the cross section shown on **1B**, where choroidal thickness was measured manually with the inbuilt caliper in the center of each ETDRS field. **1C** shows the automatically calculated average thickness and volume of the retina in the field.

All patients after pupil dilatation underwent SD-OCT imaging by the Spectralis (Heidelberg Engineering Germany, Version 1.6.4.0). Images were acquired using horizontal raster pattern scans, which were obtained via a 20 × 20-degree (5.4 × 5.4) scan field, consisting of 49 sections.Retinal thickness in the 9 Early Treatment Diabetic Retinopathy Study (ETDRS) subfields was analyzed by the RT map analysis protocol. Choroidal thickness was measured with EDI scans, in the center of each ETDRS subfield (Figure 1A). The central subfield (CSF) was a region with a diameter of 1.0 mm around the fovea, and the inner and the outer rings had diameters of 3 and 6 mm. Choroidal thickness measurement was performed perpendicular to the RPE, going from the posterior RPE edge to the choroid–scleral junction in the center of all ETRDS subfields (Figure 1B). Evaluation of OCT scans and measurement of retinal and choroidal thickness was done by an independent and masked reader. Retinal and choroidal thickness in all ETDRS fields was recorded. The mean thickness of the 3 mm (average of S3, T3, N3 and I3 fields) and 6 mm (average of S6, T6, N6 and I6 fields) rings was calculated and used for further statistical analysis.

### Statistical analysis

The statistical analysis was performed using the Statistica 11.0 software (Statsoft, Tulsa, OK, USA). Data distribution was checked by Shapiro-Wilk’s W test, which showed non-normally distributed data, hence nonparametric tests were conducted. Kruskal-Wallis one-way analysis of variance test was applied to compare mean retinal and choroidal thicknesses among the three patient groups. Wilcoxon nonparametric test was the tool for comparative evaluation between CSF and perifoveal rings. Additionally we correlated retinal thickness to choroidal thickness at different measurement points (Spearman rank correlation). Data were expressed as mean values ± standard deviation. Significance was accepted for p < 0.05. Fisher exact test was used to determine the rate of cystoid macular edema.

Receiver operating characteristic (ROC) curve was plotted by means of the Med Calc Version 12.5.0.0 software (Med Calc Software bvba, Ostend, Belgium), and the cut-off value was calculated from this curve.

## Results

The study involved 78 participants. They were put into a control group (n = 34; mean age 37.4 ± 10.6 range: 23 to 67 years), an AU group (n = 21; mean age 41.2 ± 13.25 range: 21 to 73 years) and an IMU group (n = 23; mean age 39.7 ± 13.41 range: 22 to 80 years). The mean ages of the 3 groups were not significantly different (p = 0.5838, Kruskal-Wallis test), thus the control group was considered age-matched.

### Retinal thickness

**Figure 2:**
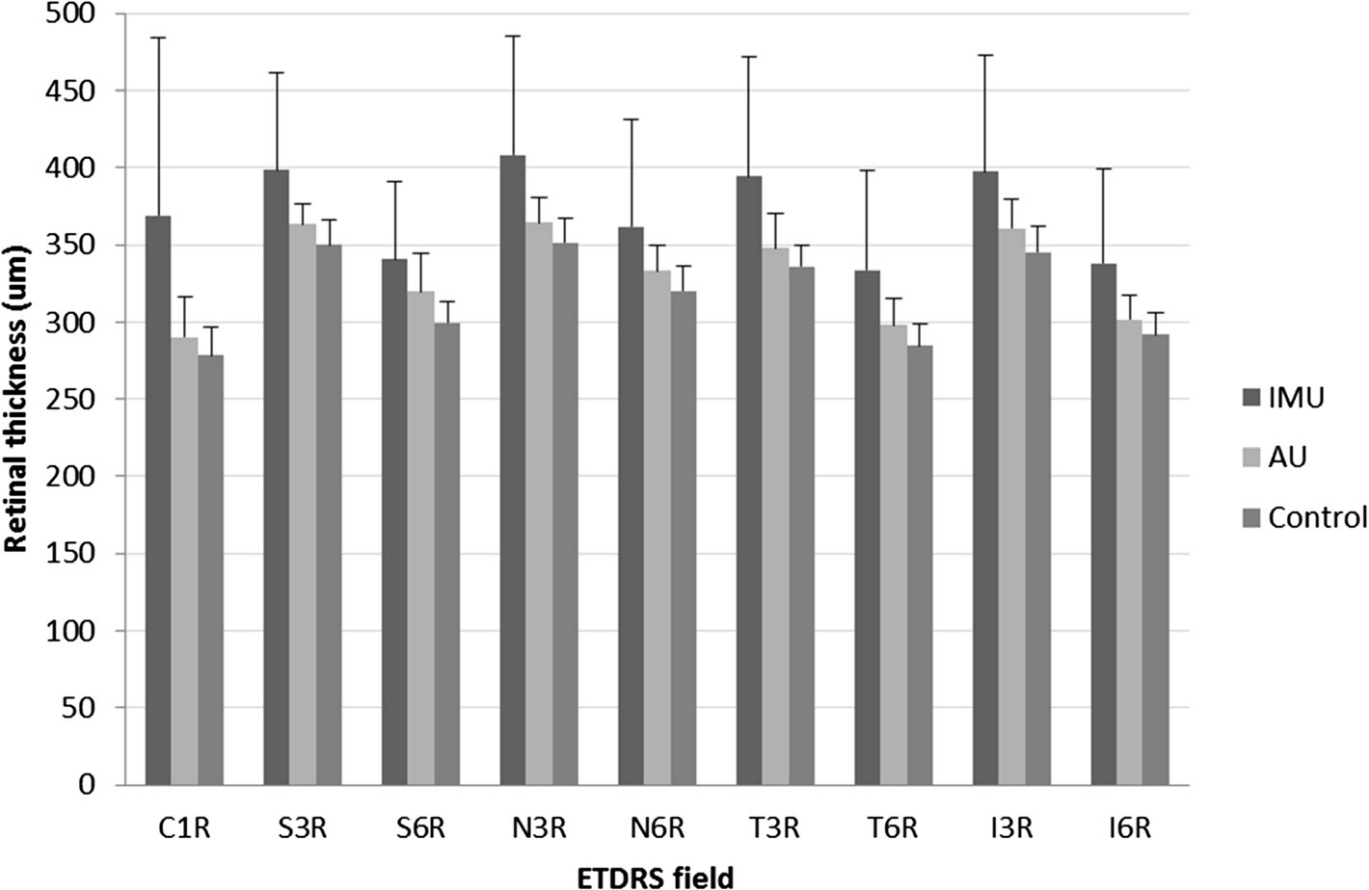
**Retinal thickness in the 9 fields (Mean ± SD).** C1R = central subfiled, S3R = superior quadrant of 3 mm ring, S6R = superior quadrant of 6 mm ring, N3R = nasal quadrant of 3 mm ring, N6R = nasal quadrant of 6 mm ring, T3R = temporal quadrant of 3 mm ring, T6R = temporal quadrant of 6 mm ring, I3R = inferior quadrant of 3 mm ring, I6R = inferior quadrant of 6 mm ring. Significant differences are detailed in Table 1.

Comparison of the mean retinal thickness at each retinal location of AU and IMU groups to the control group are shown in Figure 2. We also analyzed the retinal thickness in perifoveal concentric rings, the results and the p values for the differences between the groups are summarized in Table 1. CSF thickness was 278.55 ± 18.31 μm for the control group, 290.42 ± 26.37 μm for the AU and 368.65 ± 115.88 μm for the IMU group. There was no significant difference in the CSF thickness of the AU patients and the control group, but the CSF thickness of the IMU patients was significantly higher than in healthy individuals (p = 0.0001). The retina was considerably thicker in both the 3 mm and the 6 mm zone, in the AU and IMU groups alike if compared to the control group (Table 1).

**Table 1:** Comparison of retinal thickness and choroidal thickness among groups with Kruskal-Wallis test

	**Location**	**Thickness (Mean ± SD, μm)**			**Kruskal-Wallis p value**			
		**IMU**	**AU**	**Normal**		**Post hoc**		
						**Normal-IMU**	**Normal-AU**	**IMU-AU**
Retina	Central (CSF)	368.65 ± 115.88	290.42 ± 26.37	278.55 ± 18.31	0.0003*	0.0001*	0.617	0.0407*
	3 mm ring	390.55 ± 70.90^†^	359 ± 15.24^†^	345.41 ± 15.28^†^	0.0004*	0.0003*	0.0388*	0.7536
	6 mm ring	343.33 ± 57.29^†‡^	313.83 ± 16.63^†‡^	299.09 ± 13.82^†‡^	<0.0001*	<0.0001*	0.0171*	0.4772
Choroid	Central	303.17 ± 93.66	312.61 ± 90.35	311.94 ± 60.48	0.6082	NA	NA	NA
	3 mm ring	289.43 ± 74.5	296.46 ± 81.11^†^	297.71 ± 55.7^†^	0.6666	NA	NA	NA
	6 mm ring	261.7 ± 66.01^†‡^	277.17 ± 73.23^†‡^	285.16 ± 48.82^†‡^	0.1435	NA	NA	NA

*IMU*- Intermediate uveitis, *AU*- Anterior uveitis, *CSF*-Central subfield.

*- siginficant difference (Kruskal-Wallis test p < 0.05).

^†^Different from center (Wilcoxon test p < 0.05).

^‡^Different from 3 mm ring (Wilcoxon test p < 0.05).

*NA* = Not applicable.

Comparison of the CSF to the 3 and 6 mm perifoveal rings in each group showed that in the AU group the thickest region of the retina was the 3 mm ring, while it was the thinnest in the centrum, similar to the healthy retinas. In contrast to this in the IMU group the thickest part of the retina was the 3 mm ring but it was followed by the centrum and the thinnest region was the 6 mm ring.

ROC analysis of retinal thickness results are summarized in Table 2. In the CSF analysis was not applicable in normal-AU relation due to the lack of significant difference, while in the IMU group a substantial thickening of the retina in CSF was found.

**Table 2:** ROC analysis of retinal thickness values

		**Central (CSF)**	**3 mm ring**	**6 mm ring**
Normal - IMU	AUC	0.800	0.765	0.822
	Specificity	91.1	100	70.6
	Sensitivity	73.9	56.5	87.0
	Cut-off value (μm)	297	383	304
	p value	<0.0001	0.0003	<0.0001
Normal - AU	AUC	NA	0.659	0.750
	Specificity	NA	82.4	66.7
	Sensitivity	NA	57.1	76.5
	Cut-off value (μm)	NA	358	306
	p value	NA	0.0004	0.0002

The high sensitivity and specificity of the cut-off value received as a result of the ROC-analysis in the central retinal thickness allows us to use the 297 μm as a lower threshold for the macular edema in IMU.

*AUC* - Area under curve, *CSF*-Central subfield.

*NA* = Not applicable.

Regarding the morphology of the macular thickening only one in 21 AU patients whereas 9 in 23 IMU patients had cystoid macular edema (CME), (p = 0.007, Fisher exact test). We found central subretinal fluid (SRF) in four patients with cystoid and in two patients with diffuse type of macular edema - all 6 cases occurred among the IMU patients.

### Choroidal thickness

Central choroidal thickness, measured at the fovea was 311.94 ± 60.48 μm in control eyes, 312.61 ± 90.35 μm in the AU and 303.17 ± 93.66 μm in the IMU group. The difference was not significant between the groups in the centrum or in the perifoveal rings either (Kruskal-Wallis test, p = 0.6082) (Table 1).

**Figure 3:**
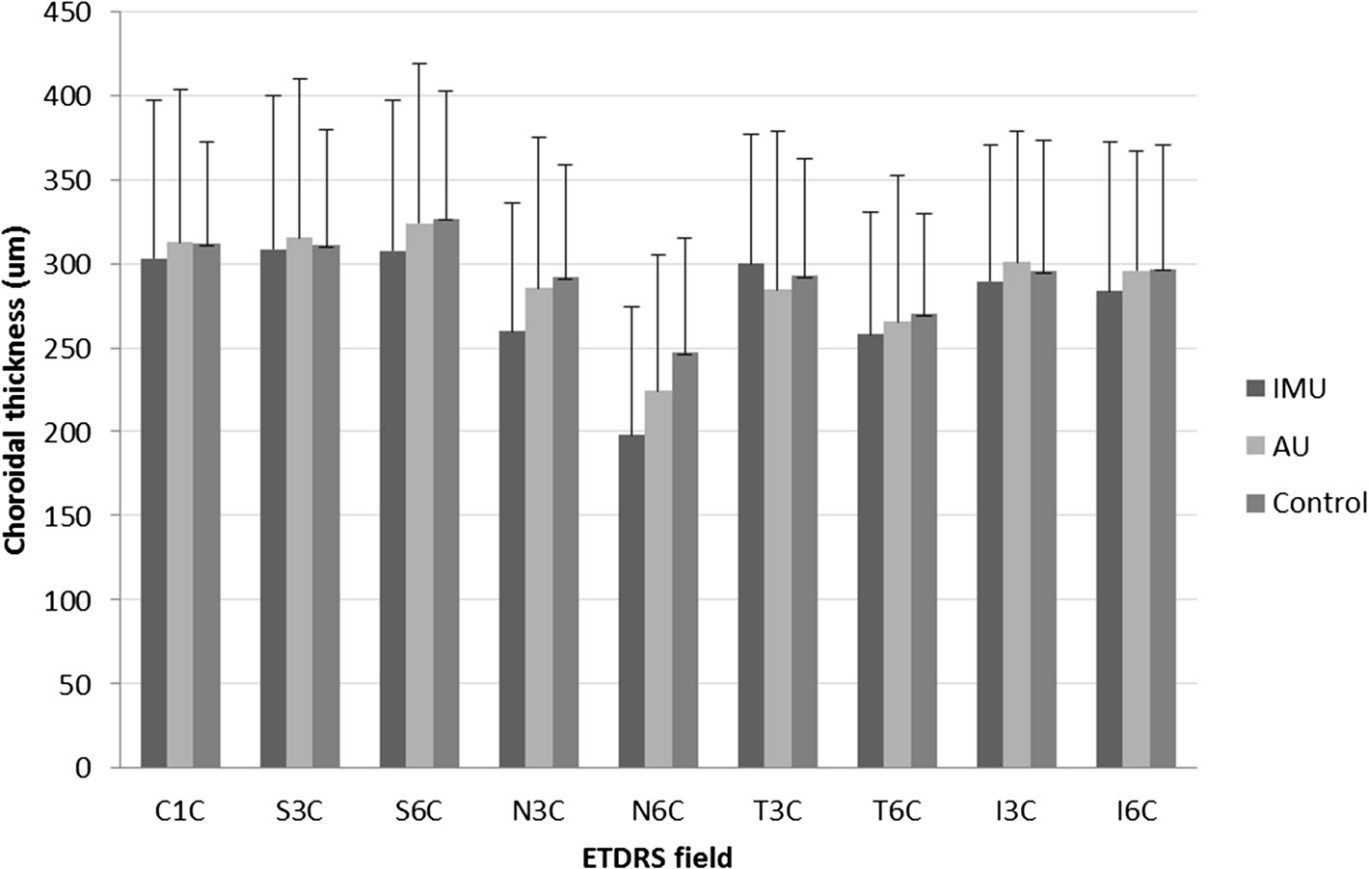
**Choroidal thickness in the 9 fields (Mean ± SD).** C1C = central subfiled, S3C = superior quadrant of 3 mm ring, S6C = superior quadrant of 6 mm ring, N3C = nasal quadrant of 3 mm ring, N6C = nasal quadrant of 6 mm ring, T3C = temporal quadrant of 3 mm ring, T6C = temporal quadrant of 6 mm ring, I3C = inferior quadrant of 3 mm ring, I6C = inferior quadrant of 6 mm ring. Significant differences are detailed in Table 1.

Comparison of the CSF to the 3 and 6 mm perifoveal rings in each group showed that the choroidal thickness in the 3 and 6 mm ring was not different from the CSF in the AU and in the control groups. However, in IMU patients both 3 mm and 6 mm rings were different from the centrum (Wilcoxon test, p < 0.05) (Table 1).Although in the IMU the choroidal thickness was significantly thinner in the nasal 6 mm quadrant than in the control group (p = 0.0364), none of the other quadrants differed significantly from each other in terms of thickness in the three groups (Figure 3).

### Correlation of retinal and choroidal structure

No significant correlation could be demonstrated between retinal and choroidal thickness in the corresponding fields, neither in the AU nor in the IMU group (p > 0.05, Spearman rank correlation).

## Discussion

Macular edema frequently accompanies AU and IMU [2]. Our study focused on the distribution of intraretinal and intrachoroidal fluid in AU and IMU according to ETDRS fields. To our best knowledge, ETDRS field based, detailed and combined evaluation of macular edema and choroidal thickness has not been described before in AU and IMU. The better understanding of the correlation between retinal and choroidal involvement in the above mentioned forms of occular inflammation could be achieved with our study.

### Retinal thickness

Recent studies using OCT showed that in AU biomicroscopically often undetected macular edema becomes visualized even by means of normal retinal morphology [4,5]. Intermediate uveitis is frequently (in almost half of the cases) accompanied by macular edema [2].

Shulman et al described that some degree of macular edema can be found even in mild anterior uveitis [5]. Our results support this observation. In contrast to this, as regards the CSF thickness we did not find significant difference in the CSF thickness between individuals with AU and age-matched controls, but the retinal thickness was substantially higher in both the 3 mm and the 6 mm rings, retaining the retinal arrangement similar to normal maculae [8]. Our results are in accordance with Castellano et al., namely that in AU the retinal thickening is present typically in perifoveal ring-like distribution [9]. This result suggests the importance of OCT examination in all types of AU, and that the assessment should especially take into account the 3 mm inner ETDRS ring values, not only CSF thickness. Interestingly in the IMU group we also found a similar architecture, i.e. the 3 mm ring with the highest retinal thickness, followed by the CSF and the relative thinnest outer 6 mm ring. On the contrary, in the AU group all three zones were significantly thicker than in the control group. While among the 21 AU patients we found only one patient with CME, nine patients out of 23 in the IMU group had CME. In agreement with the findings of other workgroups, our data show that the diffuse type of macular edema is much more characteristic of AU than of CME [4].

Another OCT feature connected to IMU is SRF which we found in six eyes and all of them were in the IMU group. This means that SRF evolved in 13% of all uveitis cases, in contrast to the higher percentages reported earlier [8,9]. This result seems to be contrasting with the observations that SRF appears in the initial phase of the inflammatory process, but we have to emphasize that not all patients had macular edema in our cohort [10].

### Choroidal thickness

Investigating the choroidal thickness in macular edema Regatieri et al described decreased choroidal thickness in patients with diabetic macular edema [11]. In contrast with this result, Xu et al concluded that diabetic macular edema did not influence the choroidal thickness, similarly to our study on uveitic macular edema [12].

Despite the choroidal involvement in uveitis, there are only few publications investigating choroidal thickness in any type of uveitis. Posterior uveitis accompanied mostly by thickening of choroidal layers, especially in acute phase [13-17].

Assumably in posterior uveitis, where the choroid is affected in inflammatory processes, choroidal thickness is influenced by many – sometimes adverse – effects. Inflammatory infiltration could potentially result in increased thickness, whereas the diminished choroidal circulation may result in reduced choroidal thickness.

This hypothesis is supported also by observations of Karampelas et al. They concluded that in 21 inactive panuveitic eyes the mean choroidal thickness was lower and hyporeflectivity in Haller’s large vessel layer was reduced. They attributed these results to choroidal ischemia and hypoperfusion [18].

It seems, that the choroidal changes of macular region in AU and IMU are less comparable to the results of measuremets of posterior uveitis, where the infiltration of the choroid is likely.

We have come to the conclusion in our control group that the mean subfoveal choroidal thickness was in the higher normal range, which may reflect the lower mean age of the participants than it is in other studies.

To our best knowledge this is the first study investigating choroidal thickness in AU and IMU. We found the choroidal thickness to be markedly thinner only in IMU in the nasal 6 mm quadrant, none of the other quadrants were significantly lower in uveitis groups than the thickness observed in normal subjects. Interestingly this is the thinnest region of the macula in normal subjects and it is also the only quadrant where the choroid considerably attenuated. It may be either a coincident or it could reflect to the vulnerable choroidal circulation of the papillomacular region.

We have demonstrated significant retinal thickening in AU and IMU, but only limited involvement of the choroid could be demonstrated. Our study did not provide sufficient evidence about the casual relationship between the microstructural changes of the macula and the choroidal structure. Our results can be interpreted in several ways. One option is that the lack of correlation can be attributed to the early phase of the inflammatory process; the other explanation is that the effect of uveitic macular edema on choroidal circulation is questionable, similar to the effect of diabetic macular edema.

The limitations of this study are the small number of the patients and the lack of follow up measurements in the chronic inflammation phase. Further limitation is the lack of available inbuilt software for the automated measurement of choroidal thickness. Normative database is not uniform, the reported normal subfoveal choroidal thickness varied from 225 μm to 311 μm [19-21], Ikuno et al have however found good interobserver reproducibility of choroidal thickness measurement [22]. Detection of the choroid-sclera border could be improved by the recently introduced swept-source optical coherence tomography, which compared with SD-OCT has greater sensitivity at scanning deep choroidal structures and the superficial retinal layers in the same image, and with longer wavelength enabling better imaging of deeper structures [23].

## Conclusion

In conclusion we can say that the retinal thickness increases in AU and IMU already in the early disease process, but choroidal thickness seems to be unaffected and not to be related to retinal thickness. In the evaluation of the macular structure in AU patients it is especially recommended to analyze the OCT results of the perifoveal region, since it provides early detection of macular edema, even in case of normal CSFT. In IMU cases either the center or the perifoveal macular regions are affected by macular edema.

Our OCT findings demonstrated that in contrast with posterior uveitis, anterior and interediate uveitis promotes fluid accumulation mainly in the retina and not in the choroid. Further development of OCT imaging technology and multimodal imaging could provide more detailed insight in the pathomechanism of macular strutural changes in different types of uveitis.

## Competing interests

The authors declare that they have no competing interests.

## Authors’ contributions

ZG recruited the patients, wrote the manuscript, participated in study design. KK carried out the measurements. HK helped in formatting, language, reviewed the literature. JN participated in study design. OM participated in measurements. MR performed the statistical analysis and helped to draft the manuscript. All authors read and approved the final manuscript.

## Pre-publication history

The pre-publication history for this paper can be accessed here:

http://www.biomedcentral.com/1471-2415/14/103/prepub
